# Acute Aortic Dissection Mimicking STEMI in the Catheterization Laboratory: Early Recognition Is Mandatory

**DOI:** 10.1155/2012/367542

**Published:** 2012-09-27

**Authors:** Alessio Arrivi, Gaetano Tanzilli, Paolo Emilio Puddu, Giovanni Truscelli, Marcello Dominici, Enrico Mangieri

**Affiliations:** ^1^Division of Cardiology, Santa Maria University Hospital, 05100 Terni, Italy; ^2^Department of Cardiovascular, Respiratory, Nephrological, Anesthesiological and Geriatrical Sciences, Sapienza University, 00161 Rome, Italy

## Abstract

Coronary malperfusion due to type A aortic dissection is a life-threatening condition where timely recognition and treatment are mandatory. A 77-year-old woman underwent an acute evolving type A aortic dissection mimicking acute myocardial infarction. Two pathophysiologic mechanisms are discussed: either thrombosis migrating from a previously treated giant aneurism of proximal left anterior descending or a local arterial complication due to left main stenting. Recognition of these occurrences in the catheterization laboratory is important to look immediately for surgery.

## 1. Case Report

**Figure 1:**
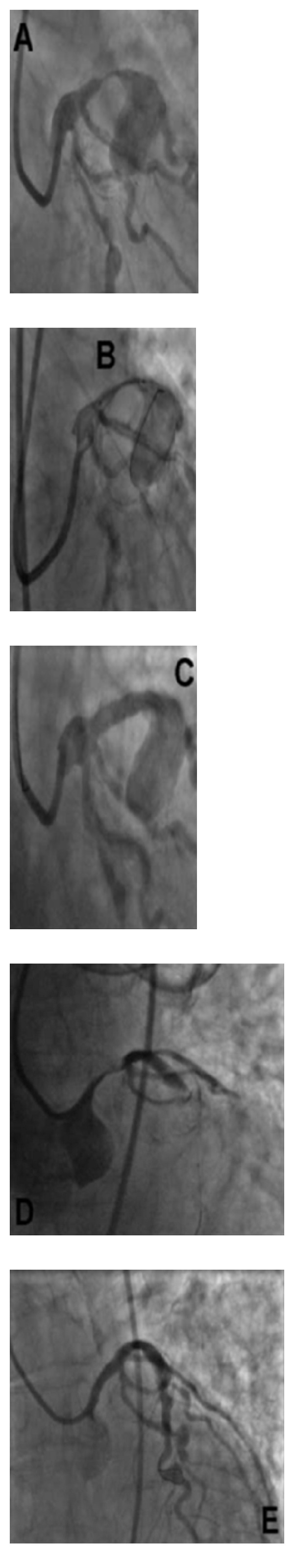
Angiographic projections in (A) (23°RAO and 21°CAU): giant, calcified, aneurism of proximal LAD, and coronary subocclusion immediately proximal to the sac; (B) (21°RAO and 7°CAU): PCI with stent placement in LAD; (C) (21°RAO and 15°CAU): angiographic result after stenting of LAD; (D) (3°LAO and 1°CRA): subocclusion of the left main coronary artery (LMCA) with patency of the bare metal stent previously implanted in the proximal tract of the LAD and closure of the coronary aneurism; (E) (17°LAO and 4°CAU): angiographic result after primary PCI. LAO: left anterior oblique; RAO: right anterior oblique; CRA: cranial; CAU: caudal.

A 77-year-old woman presented complaining from the sudden onset of acute retrosternal pain radiating to the left arm. Three months earlier, the patient was referred to our laboratory for a giant, calcified, aneurism of proximal LAD and coronary subocclusion immediately proximal to the sac (Figure 1(A)) and percutaneous coronary intervention (PCI) was performed using a 3.5 × 14 mm bare metal stent (BMS) (Figures 1(B) and 1(C)).

**Figure 2:**
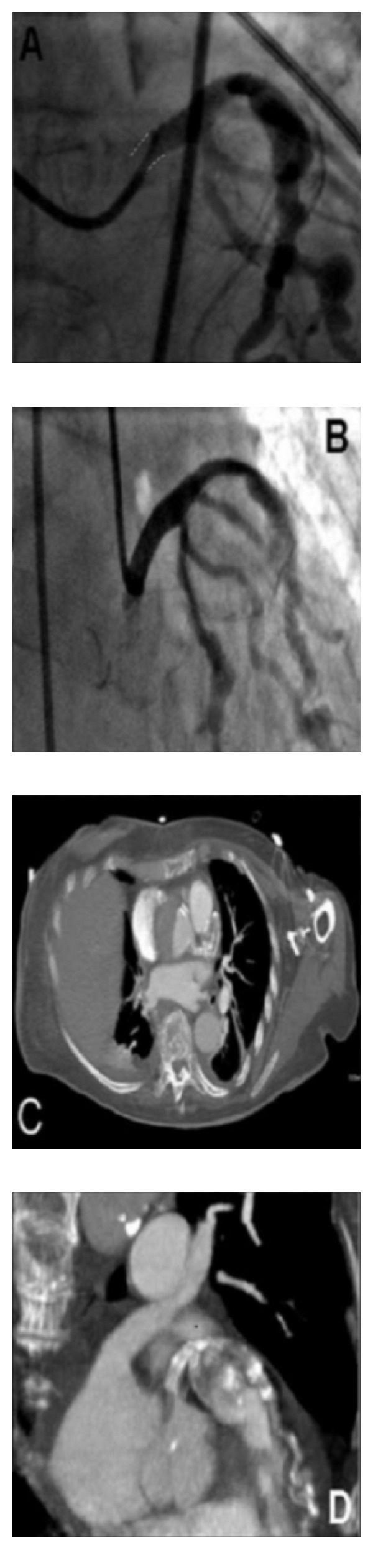
Angiographic projections in (A) and (B); (A) (35°LAO and 24°CRA): significant length reduction (dashed lines) of the LMCA; (B) (28°RAO and 6°CAU): PCI with implantation of 4.5 × 13 mm BMS on the proximal tract of the LMCA overlapping with the previously implanted stent. CT scan in (C) (axial scanning) and (D) (3D volume rendering reconstruction) whereby aortic dissection involving the LMCA ostium and thrombosis of the coronary aneurism are seen. LAO: left anterior oblique; RAO: right anterior oblique; CRA: cranial; CAU: caudal.

During the current hospitalization, she had severe hypotension (70/50 mmHg) with heart rate of 63 beats/min. The ECG showed diffuse ST segment depression and elevation in lead aVR, suggesting an acute myocardial infarction (AMI) due to possible occlusion of the left main coronary artery (LMCA). Anteroposterior chest X-rays showed no significant widening of the mediastinum. The patient was under double antiplatelet therapy with aspirin 100 mg and clopidogrel 75 mg. Transferred to the laboratory for primary PCI, there was the evidence of subocclusion of the LMCA with patency of the BMS previously implanted in the LAD and closure of the coronary aneurism (Figure 1(D)). LMCA was then stented using a 3.5 × 9 mm BMS (Figure 1(E)). Because of recurrent episodes of ventricular tachycardia, a control angiogram soon after the procedure showed a significant length reduction of LMCA, just prior the implanted stent (Figure 2(A)). Thus, in overlap with the first stent, a second one (BMS 4.5 × 13 mm) was applied in the proximal tract of LMCA (Figure 2(B)).

Back in the ward, the patient underwent transthoracic echocardiography whereby a double outline was seen at the level of ascending aorta. A successive CT scan clearly showed dissection of the ascending aorta starting near the LMCA (Figures 2(C) and 2(D)). The patient suddenly died before surgery could be undertaken. No autopsy was performed.

## 2. Discussion

The correct diagnosis of an acute Stanford type A aortic dissection is a “dare” for the physician. The mortality rate of this event is high, around 68% in the first 48 hours [1–3]. This report shows the difficulty in the detection of an aortic dissection, when clinical symptoms, patient's history, and initial instrumental examinations address towards the diagnosis of cardiogenic shock due to AMI. Previous LAD stenting and the presence of a coronary aneurism increased the pretest probability for acute coronary syndrome. AMI due to AAD is reported in about 3% of cases and is associated with high mortality rate. The right coronary artery is more often affected than the left with resulting acute inferior ischemia [4, 5]. However, previous ischemic heart disease might be a confounder concurring to misleading diagnosis. This points out the importance of performing an appropriate diagnostic workup, including pulse examination, cardiac auscultation, and fast echocardiogram in all cases of suspected acute coronary syndromes (ACSs), including those that “a priori” may have the highest probability because of prior PCI. Diagnostic coronary angiography is often necessary, but echo and CT may more appropriately exclude AAD and/or help in the differentiation from other conditions [6]. All too frequently patients are rushed into the catheterization laboratory in order to reduce precoronary time, but sometimes this can translate into useless and/or dangerous races.

LCA collapsing might have followed to coronary malperfusion subsequent to intermittently obstructing intimal aortic flap. AAD due to prior LAD aneurism and a likely endothelial dysfunction should be suspected. However, from the angiographic point of view, it is difficult to recognize AAD when coronary angiography is performed during primary PCI and the guide catheter easily engages the coronary ostium without any staining of the aortic wall during dye injection. Neither were any movements seen relating to intimal flap at the monitor level during catheterization, likely because the AAD began as a localized one near the LMCA ostium. A further possibility was a periprocedural complication due to implantation of stents in a very calcified vessel (as seen in Figure 2(D)) ending-up as AAD.

Thus, in presence of sudden LMCA occlusion with the typical image of “caliber reduction,” which was shown, with high but late accuracy by CT scan detecting false lumen extension beyond the coronary ostium, AAD should be suspected and surgery looked for immediately.
